# Smarter than Others? Conjectures in Lowest Unique Bid Auctions

**DOI:** 10.1371/journal.pone.0122923

**Published:** 2015-04-07

**Authors:** Cancan Zhou, Hongguang Dong, Rui Hu, Qinghua Chen

**Affiliations:** 1 School of Systems Science, Beijing Normal University, Beijing, People’s Republic of China; 2 Beijing Higher Education Press, Beijing, People’s Republic of China; Tianjin University, CHINA

## Abstract

Research concerning various types of auctions, such as English auctions, Dutch auctions, highest-price sealed-bid auctions, and second-price sealed-bid auctions, is always a topic of considerable interest in interdisciplinary fields. The type of auction, known as a lowest unique bid auction (LUBA), has also attracted significant attention. Various models have been proposed, but they often fail to explain satisfactorily the real bid-distribution characteristics. This paper discusses LUBA bid-distribution characteristics, including the inverted-J shape and the exponential decrease in the upper region. The authors note that this type of distribution, which initially increases and later decreases, cannot be derived from the symmetric Nash equilibrium framework based on perfect information that has previously been used. A novel optimization model based on non-perfect information is presented. The kernel of this model is the premise that agents make decisions to achieve maximum profit based on imaginary information or assumptions regarding the behavior of others.

## Introduction

Auctions, which represent a typical human economic behavior, have a long history: records indicate that auctions were held as early as 500 B.C., and they have evolved into various kinds of new auctions ^[^ ^1^ ^]^. Recently, a new type of auction, the lowest unique bid auction (LUBA), has been gaining in popularity throughout the world [2–4]. Unlike traditional types of auctions such as English or Dutch auctions, this type of reverse auction allows the winner to gain an expensive object at an extremely low cost. In addition to generating enthusiasm, the bid-distribution characteristics and mechanism have attracted the attention of many researchers. Meanwhile, the lowest unique positive integer game (LUPI) and related studies have also garnered considerable interest [5,6]. In fact, the stable distributions of both LUBA and LUPI are the same, with higher bid probabilities at lower prices and lower probabilities at higher prices [3–6]. However, it is clear that players in LUBA and LUPI are faced with the same strategic conflict in attempting to choose bids that are both low and unique [5].

To explain the formation of this distribution, several models have been proposed. Rapoport, Otsubo, Kim & Stein [3] have constructed symmetric mixed-strategy equilibrium solutions and then tested them in a series of experiments in which the number of bidders and the size of the strategy space were varied. Houba, van der Laan & Veldhuizen [4] have concluded that the symmetric NE (Nash equilibrium) with the lowest expected gains is the maximin in a symmetric strategy; this criterion allows for computation using a mathematical program. Pigolotti, Bernhardsson, Juul, Galster & Vivo [7] have used a grand canonical approach to derive an analytical expression for the equilibrium distribution of strategies. They then studied the properties of the solution as a function of the mean number of players and compared their results against a large data set of internet auctions. Very recently, Zhao, Chen & Wang [8] have developed a model in which it is assumed that agents prefer to bid on the price at which the probability of winning is highest. However, almost all such studies have been based on the symmetric Nash equilibrium.

Upon further consideration, the reliability of this paradigm of the Nash equilibrium based on perfect information is called into question. First, equilibrium models based on the assumption of perfect information can only reproduce a monotonically decreasing trend in bid probability as the bid price increases [4,7,8]. They cannot provide a good explanation for the increasing regime observed in the empirical data, as later discussed. Moreover, under the assumption of the symmetric Nash equilibrium condition with perfect information, each agent will tend to receive only the average payoff. The likelihood of winning relies solely on luck or random chance, which is unacceptable for actual participants with fervent desires to win. In fact, any agent who participates in such an auction does so with the expectation of receiving a higher payoff than the other participants.

Several valuable essays have provided the means of formulating certain consistent assumptions. Camerer, Ho & Chong [9] have developed a cognitive hierarchy (CH) model for two types of games: smarter participants are ranked at higher levels in the cognition system and make decisions based on others' decisions, but others do not have access to this agent's information when they make their decisions. Ostling, Wang, Chou & Camerer [5] concludes that some of the deviations from equilibrium in the Swedish lowest unique positive integer (LUPI) game can be rationalized by a cognitive hierarchy model. Rothkopf [10] has investigated this topic in the context of decision theory. He has suggested that agents must optimize their choices of bids against their assessed or calculated previous knowledge of the probability distribution. In these studies, it is supposed that every agent believes that he is at a deeper cognitive level than the others. This belief that he is “smarter” helps him to revise his strategy to gain more profit than others. Although this psychological factor holds true in every auction, it should be noted that each agent must estimate or guess his competitors’ decisions because of lacking information.

This paper presents the LUBA bid distribution and proposes an optimization model based on non-perfect information. The kernel of this model is the premise that each agent performs the same optimization based on some imaginary information or assumptions regarding the behavior of the others. The model fits real LUBA distributions very well, and this paradigm seems to be more acceptable and reliable than that based on the symmetric Nash equilibrium. The structure of the paper is as follows. In the next section, we introduce our empirical data sources and the features of the distributions that we have identified. Then, the new model is proposed and used to reproduce the empirical data. The effects of the two model parameters are subsequently discussed in the following sections. In the final section, we present our conclusions.

## Data Sources and LUBA Bid Distributions

### Data Sources

The data analyzed in this paper were downloaded from www.auction-air.com and www.uniquebidhomes.com, which are located in different countries and host bids based on different currency units, the pound and the dollar, respectively. Agents can bid only in integral numbers of pounds on the first website but can bid in any amount, even only a few cents, on the second one.

The full record of every completed item auction is available on these websites. We chose 6 different groups of data from these two websites. Each group includes auctions of same-valued items with nearly identical bid times. The data for groups (a) to (c) were downloaded from www.auctionair.com. The data for groups (d) to (f) were obtained from www.uniquebidhomes.com. These items vary from mobile phones to digital cameras at different values *v* (from $199 to £5900), and the number of bids *N* also vary (from 160±10 to 525±5). We list the general information regarding these auction items in Table 1. Let us consider item (a), a type of Canon digital camera, as an example. £699 is the average market price of this item. Thus, all bid prices can be expected to be lower than this value. The value listed in the column Bids NO. (*N*), 199, indicates that there were 199 agents bidding (in the case of Item (b), the entry 193±7 indicates that the maximum number of bids was 200 and the minimum number was 186). The value of 16 entered in column Rounds(R) indicates that we chose 16 individual auctions with the same items and same number of participating agents. The final column, Win bids, contains the historical winning prices.

**Table 1:** Information of real LUBA.

Item		Value(*v*)	Bids NO. (*N*)	Rounds(*R*)	The lowest bid	Win bids
(a)	Canon digital camera	£699	199	16	£1	£7,15,20,24,35,…
(b)	42" Plasma from LG	£2000	193±7	8	£1	£2,10,15,31,43,…
(c)	7 nights for 2 people at the 5-star Taj Exotica	£5900	525±5	5	£1	£5,15,26,49,60
(d)	Nintendo DS Lite	$199	160±10	3	$0.01	$0.25,0.3,0.35
(e)	Apple ipod touch	$529	240±10	4	$0.01	$0.15,0.35,0.37,0.38
(f)	$1000 cash	$1000	310	6	$0.01	$0.2,0.57,0.6,0.75,0.81,0.84

### LUBA Bid Distributions

**Fig 1:**
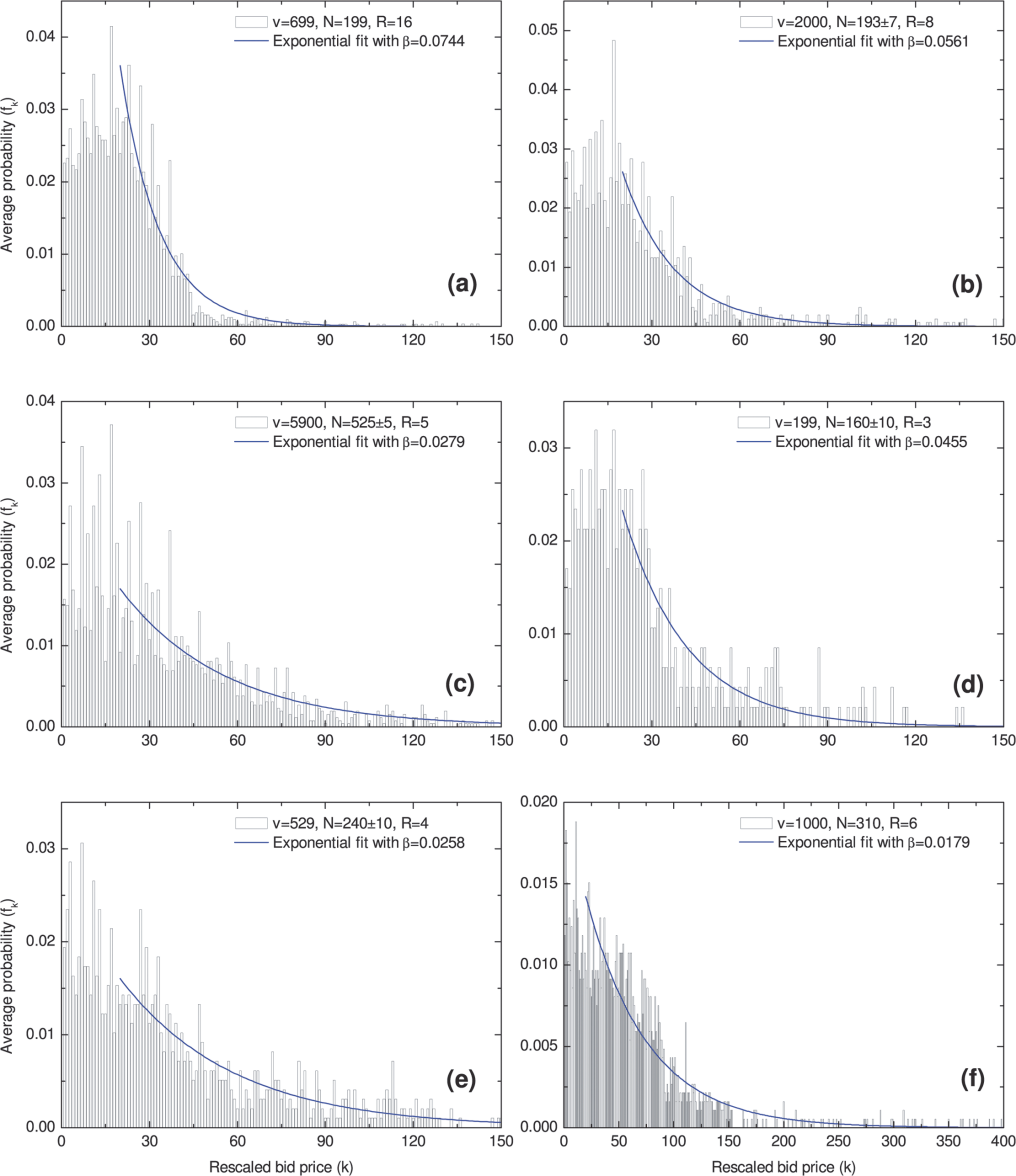
The bid distributions. The data presented in (a), (b), and (c) were obtained from www.auctionair.com, and the data in (d), (e), and (f) were obtained from www.uniquebidhomes.com. In each panel, the average calculated probability is represented by the histogram. The blue line is drawn to illustrate the exponentially decreasing probability at higher prices.

The bid distributions of these 6 groups of LUBAs are shown in Fig 1. It is clear that more people bid at lower prices than at higher prices, despite some statistical fluctuations. Very similar bid distributions are observed for different LUBAs with different item values, different agents, and different numbers of bids. The bid-price distributions have very low expectation values and long tails at higher bid prices, consistent with the low-cost-but-high-profit expectation.

However, an increasing tendency in the bid-price distribution at lower prices is also clearly evident in many cases, causing the overall bid distributions to appear as inverted-J curves. For example, for item (a), the highest probability is at a price of £17. At the price lower than £17, the probability increases as the price increases. Then, the probability begins to decrease with increasing price after approximately £17. For item (b), the turning point is also at £17. This inverted-J bid distribution can be easily understood: agents wish to spend as little as possible while avoiding making the same bid as anyone else [5], so they tend to bid at slightly higher prices.

The approximately exponential decrease observed in the tail is another significant characteristic that deserves study. The bid probability decreases with an approximately exponential tendency, *f*(*k*) ∝ *e* ^*−βk*^ [7], in the tail, as shown in Fig 1 and Table 2.

**Table 2:** The fitting with exponential functions.

Item	*β*	RSS	*R* ^2^	KS
(a)	0.0744	8.7188E-4	0.9073	0.0210
(b)	0.0561	6.9232E-4	0.8954	0.0173
(c)	0.0279	9.6616E-4	0.8378	0.0177
(d)	0.0455	8.8187E-4	0.8620	0.0280
(e)	0.0258	6.0335E-4	0.8708	0.0172
(f)	0.0179	4.7402E-4	0.8925	0.0357

## Model and Fitting

Why do the distributions emerge in this way? Scholars have introduced various models to attempt to fit these data. However, most of them fail to reproduce either the exponentially decreasing tendency or the increasing portion of the distribution. In this section, we present our model and compare it with the empirical data.

### Model

We reiterate the basic rules of LUBA as follows:

- The value of the item *v*, the number of bids *N*, the minimum bid time and other constraints are known to all agents during the entire auction process.
- Every agent is required to pay an entry fee *c*(c ≥ 0) before bidding.
- Each player offers a bid price ($k\in \left[\underline{b},\bar{b}\right]$, which is chosen from among discrete numbers (sometimes integers), based on the players’ psychology. Generally, $\underline{b}$ is equal to 0.01 or 1 (this is determined by the adjudicators of the auction), and $\bar{b}$ is often equal to *v*.
- The winner is the agent who has bid the lowest among all unique bids after the number of bids reaches *N*.

Before the construction of our model, we should clarify certain points:

- There is no functional difference among currency units.Different kinds of currents and different smallest current units do not impact the behavior of biding. Because all the players are not faced with the price of economic meaning, but the vacant positions that from left to right in turn (corresponding to the price from small to large). They consider how to make strategies so that they could get the position only themselves and make their possessions become left as far as possible (The most left position that occupied by the only person is the winning position.). No matter what the correspondence between the original vacant positions and the prices likes, we can make our discussion by using integer to code the vacant positions. And we also can easily convert a decimal number into an integer by multiplying by 100. Without loss of generality, we discuss only integer bids in this paper.
- The entry fee is irrelevant to the bid distribution. The entry fee is simply a condition determining whether an agent decides to join the auction. Once the agents have paid the fee and entered the auction, all agents are the same regardless of the previously paid cost. It is not necessary to consider the entry fee in our model.
- It is necessary to construct the model in a non-symmetric framework, as a symmetric framework poses an irreconcilable contradiction to an inverted-J-shaped bid distribution. If we suppose a bid probability distribution based on the symmetrical Nash equilibrium that contains an increasing regime, we can identify two points *k*_1_ < *k*_2_ such that the bid probability will be *f*(*k*_1_)<*f*(*k*_2_). Given this scenario, a representative agent would be more likely to bid at *k*_1_ rather than *k*_2_ when the other agents do not change their strategies because this change leads to a higher profit expectation, both by avoiding conflict with others and because a smaller number is chosen. Therefore, this state cannot be stable. For the inverted-J-shaped bid distribution to emerge, each agent must believe himself to be different from the others and perform his optimization based on his own estimation of the likely behavior of the others.
- Multiple bids from one agent can be approximately regarded as multiple different agents each bidding once. The tiny difference between these scenarios is that different participants may offer same price in the game, whereas a single rational agent will not bid more than once at the same price. In reference [7] and its supplementary material, the authors proved that the introduction of a small number of multiple bids per agent does not significantly alter the equilibrium strategy. In this paper, we assume that each agent can bid only once and that the number of bids is equal to the number of players.

We now begin to build our model. We assume that there are *N* agents in the game. The value of the item, *v*, is known to all agents. Each agent bids only once during the process. The bid price is restricted to fall between 1 and *v*.

We consider a representative agent who will make his own decision to maximize his chance of winning after determining the others’ strategies. This model is based on the Poisson distribution because of the large number of agents and the defined limits on the probability. The probability that there are no winners in the price range 1 to (*k* – 1) is

$$w(k)=\prod_{j=1}^{k-1}\left[1-\lambda \left(j\right)e^{-\lambda \left(j\right)}\right]$$ (1)

This means that the representative agent believes that either no one or more than one person will bid at prices less than *k*. Here, *λ*(*j*), the only parameter, represents the imaginary strength of the others’ bidding at price *j*. With no one bidding on price *k*, the probability of *k* being a winning number is

$$u(k)=e^{-\lambda \left(k\right)}\prod_{j=1}^{k-1}\left[1-\lambda \left(j\right)e^{-\lambda \left(j\right)}\right]$$ (2)

Here, *e* ^−*λ*(*k*)^ is the probability that no one will bid on price *k* in a Poisson process with the parameter *λ*(*k*). In this case, this agent would win if he were to bid at price.

The representative agent will choose his own mixed strategies after considering others’ decisions. The premise is that the larger the win probability *u*(*k*) is, the larger the bid probability *f*(*k*) will be. This relation is defined as follows:

f(k)∝u(k) (3)

This is a reasonable rule that has been applied to researches in many areas, such as complex networks, physics, and biology [11–13].

From (2) and (3), we obtain

$$f(k)\propto e^{-\lambda \left(k\right)}\prod_{j=1}^{k-1}\left[1-\lambda \left(j\right)e^{-\lambda \left(j\right)}\right]$$ (4)

Following the concept of cognitive levels, we assume that one agent will believe that the others will follow an exponential distribution:

$$\lambda (j)=\lambda Ce^{-\alpha j}\;\alpha >0$$ (5)

This is a monotonically decreasing function. The exponential distribution is subject to the constraint $\sum_{k=1}^{v}Ce^{-\alpha k}=1$, which is entirely determined by *α* because *ν* is given. In (5), *λ* is the number of players assumed by the representative agent.

This type of decision function is derived from a previously published discussion of logit equilibrium and quantal response equilibrium [14,15]. The authors, Anderson, Goeree & Holt [16] obtained a choice density proportional to an exponential function of expected payoffs: $f(x)=\frac{\exp(\pi^{e}(x)/\mu )}{\sum \left(\exp(\pi^{e}(x)/\mu )\right)}$, where *π*(*x*) represents the profit expectation and we set *μ* = 1 and *e* = 1.

Combining (4) and (5), we obtain

$$\frac{f(k+1)}{f(k)}=e^{-\lambda C\left[e^{-\alpha \left(k+1\right)}-e^{-\alpha k}\right]}\left[1-\lambda Ce^{-\alpha k}e^{-\lambda Ce^{-\alpha k}}\right]$$ (6)

This is a recursive equation that is entirely and exactly determined by the two exponents *λ* and *α*. If *λ* and *α* are fixed, once the original probability *f*(1) is given, all other probabilities *f*(*k*) will be determined. Because of the condition, *f*(1) will determined automatically, allowing all *f*(*k*) in Equation (6) to be precisely deduced.

### Model Fitting

**Fig 2:**
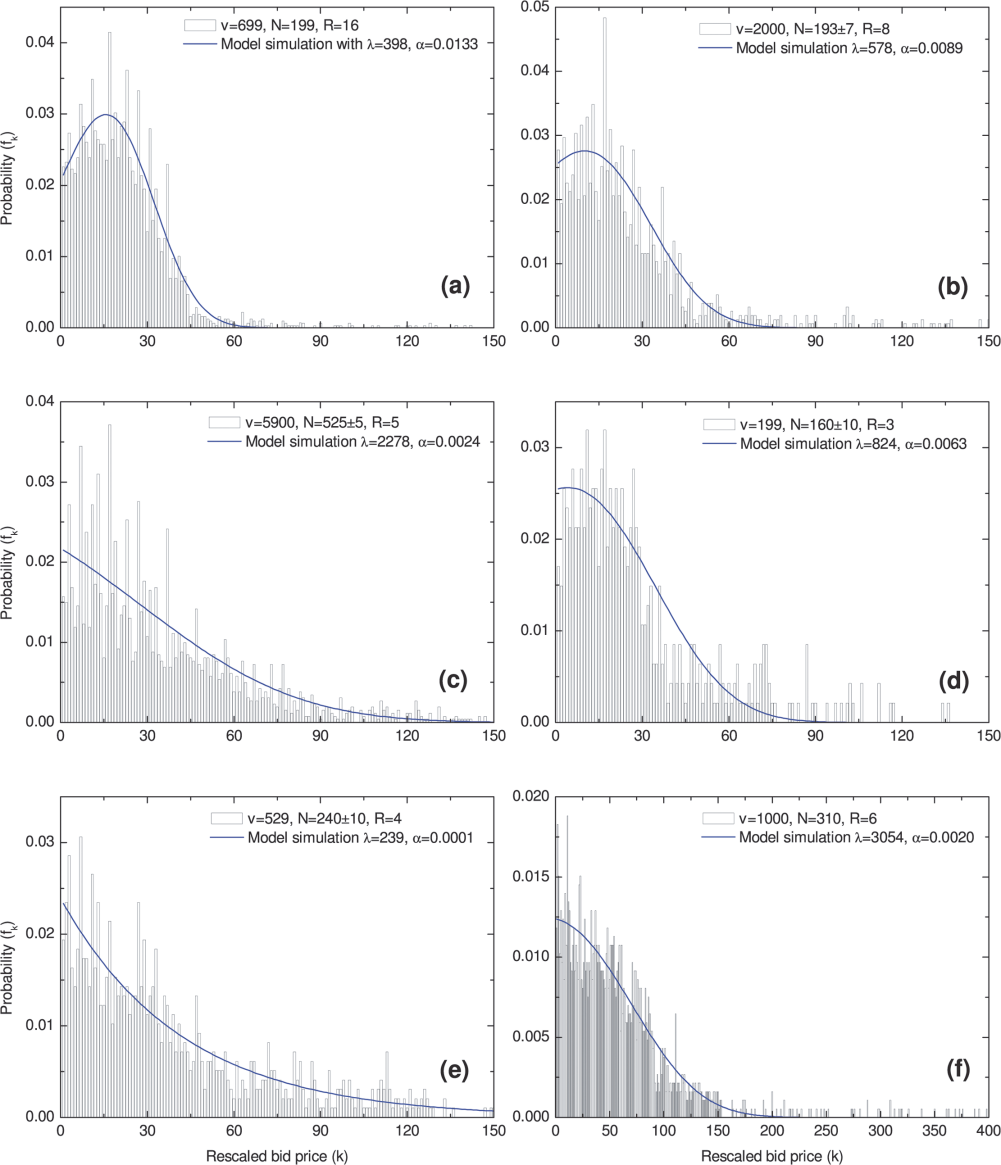
Fitting the empirical data with the model. We used Equation (6) with various parameters to fit all 6 groups of empirical data. The blue line is our model simulation, the histogram reflects the real bid distributions.

We used Equation (6) with various parameters to fit all 6 groups of empirical data investigated in this study based on least square method. In Fig 2 and Table 3, it is demonstrated that the model can predict the tendencies of real bid distributions very well. In Fig 2 (A), 2 (B) and 2 (D), our theoretical model clearly presents inverted-J curves, and it is consistent with the real trend of the actual distribution, i.e. the initial increasing regime and the later decreasing regime exactly.

**Table 3:** The Parameter Estimates and the Goodness of Fit.

Item	*λ*	*α*	RSS	R^2^	KS
(a)	398	0.0133	0.0010	0.9581	0.0229
(b)	578	0.0089	0.0018	0.9139	0.0693
(c)	2278	0.0024	0.0023	0.8438	0.0536
(d)	824	0.0063	0.0017	0.9048	0.1078
(e)	239	0.0001	0.0011	0.9140	0.0221
(f)	3054	0.0020	0.0004	0.9496	0.0259

The parameter estimates and the goodness of fit are listed in Table 3. The deviation between the fit and the true curve can be quantified by the residual sum of squares (RSS) and the Kolmogorov–Smirnov (KS) statistic [17,18]. The large *R* ^2^ values and small RSS and KS values imply that our model can fit the true distributions very well.

## Discussion of the Model

### Results of varying parameters

In our model, there are three parameters: *λ*, *α* and *ν*. Here, we discuss their effects on the bid distributions.

*λ* represents the imagined number of players. The larger *λ* is, the more agents are believed to be involved in the auction.

**Fig 3:**
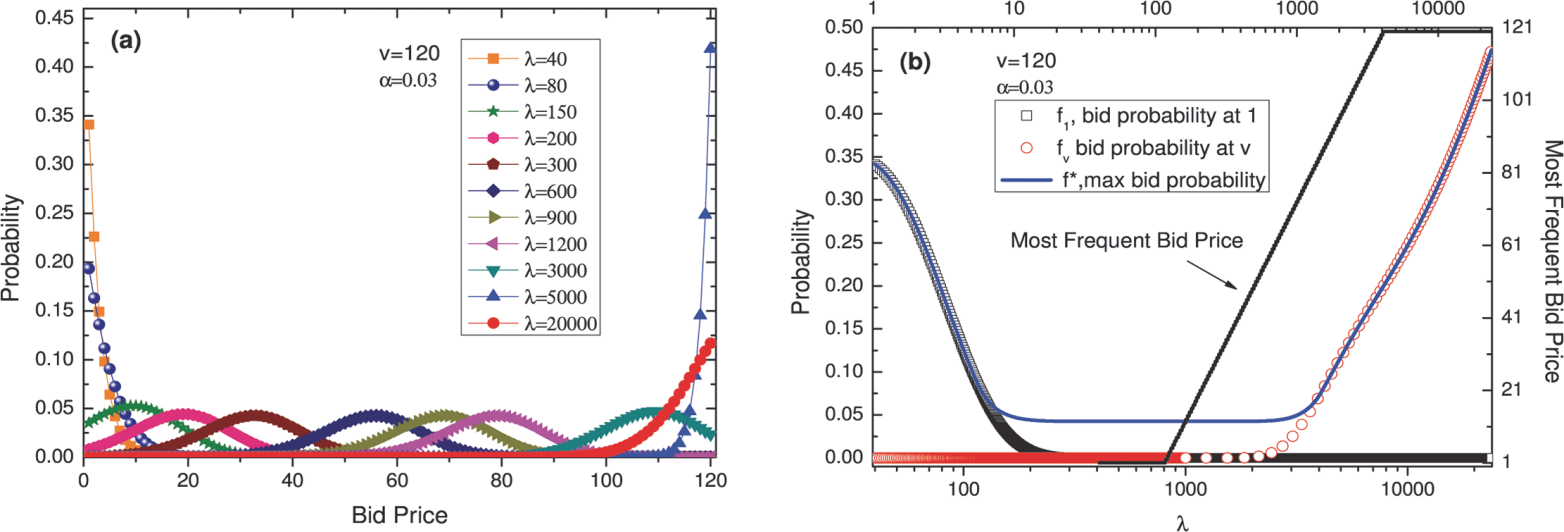
The effect of varying *λ*. With *ν* = 120 and *α* = 0.03, (a) the distribution changes like this when *λ* is varied; (b) the distribution of bid probability at 1 (quadrate line), bid probability at *ν* (red circular line), max bid probability (blue line), and most frequent bid price (black line).

Fig 3 illustrates how the distribution changes when *λ* is varied for fixed values of *ν* = 120 and *α* = 0.03. When *λ* is very small (*λ* ≤ 117), the distribution is a monotonically decreasing curve. The participation of a small number of participants relative to the price range will mean that it is more likely that no one else will bid at any particular given price, so agents will tend to bid at lower prices. When *λ* is extremely large (*λ* ≥ 4055), the distribution is a monotonically increasing curve. The price range that can be selected is too narrow compared to the number of participants, leading to concern that there may be very few prices available at which no other agent will bid. However, this scenario does not exist in practice. When *λ* is moderate, the bid distribution exhibits a peak and an inverted-J shape. According to Fig 3 (B), as *λ* increases, the location of the peak shifts logarithmically toward higher prices at an ever-decreasing rate. We also observe that the height of the peak first decreases and then increases. In the approximate *λ* range of 300 to 1600, the height of the peak stabilizes because the peak position is far from the border (between 33 and 89); therefore, the boundary has very little effect on the distribution, and the effect of varying *λ* is merely a translation of the distribution.

**Fig 4:**
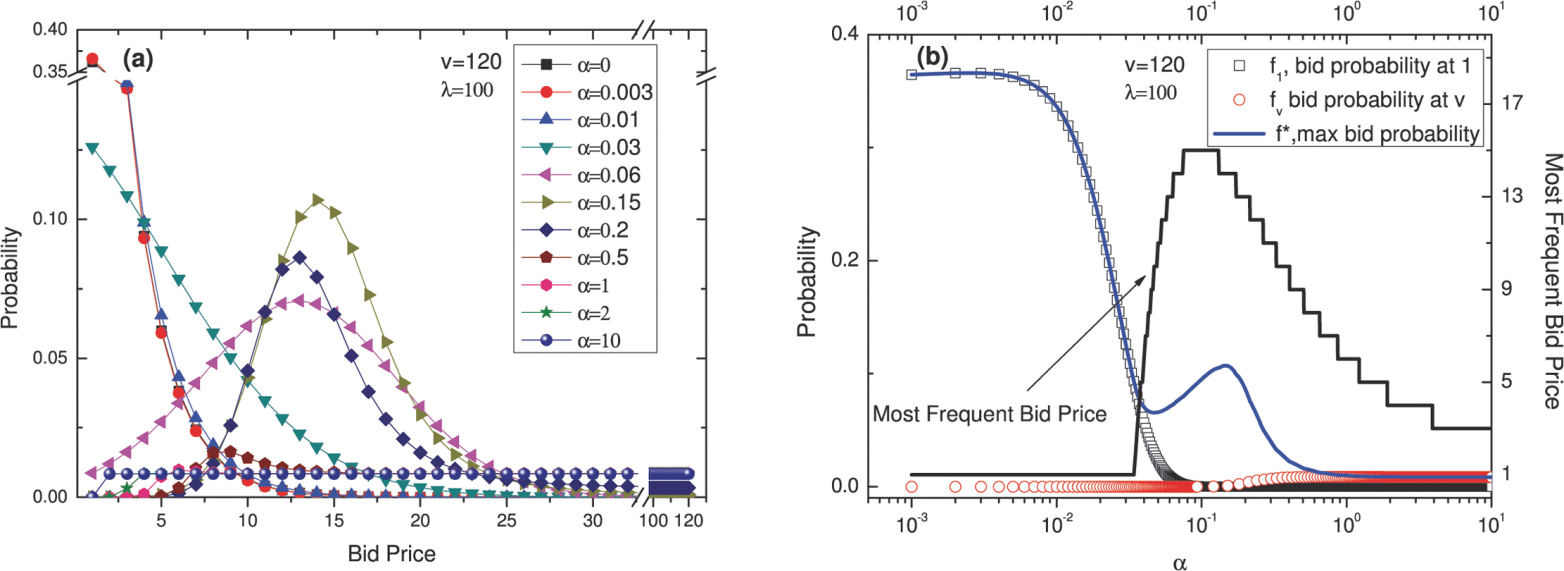
The effect of varying *α*. With *ν* = 120 and *λ* = 100, (a) the distribution changes like this when *α* is varied; (b) the distribution of bid probability at 1 (quadrate line), bid probability at *ν* (red circular line), max bid probability (blue line), and most frequent bid price (black line).

*α*, the parameter in function (5), represents the agent’s belief regarding others’ willingness to choose smaller numbers. Fig 4 presents the changes in the appearance of the distribution caused by varying *α*. When *ν* = 120 and *λ* = 100, if *α* is small (*α* ≤ 0.034), then an individual agent will imagine that others will bid at every price with nearly the same probability, and he will tend to choose low prices; as a result, a bid of 1 will have the greatest probability. As *α* increases, individuals will believe that the others will tend to focus on low prices and that bidding at very low prices will therefore incur a greater likelihood of conflicting with others; as a result, there will be a greater willingness to choose higher prices. However, as *α* increases yet further, individuals will believe that others are also likely to assume that choosing higher prices will allow them to avoid conflict with other agents, leading to vacancies at lower prices; as a result, bidding will shift toward lower prices once again.

**Fig 5:**
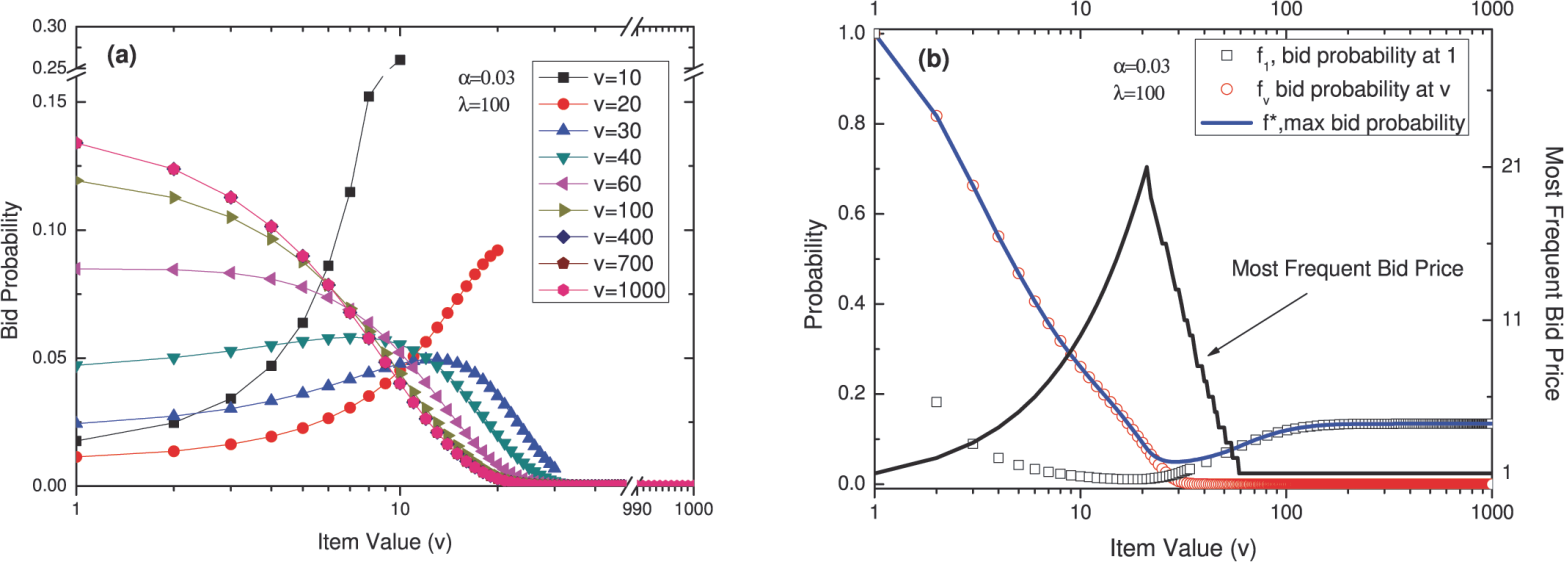
The effect of varying *ν*. With *α* = 0.03 and *λ* = 100, (a) the distribution changes like this when *ν* is varied; (b) the distribution of bid probability at 1 (quadrate line), bid probability at *ν* (red circular line), max bid probability (blue line), and most frequent bid price (black line).

The final parameter is the item value. Given *α* = 0.03 and *λ* = 100, when is too small, it will be assumed that other bids will be clustered at low prices, so individuals will tend to bid at higher prices, with a strong likelihood of choosing the highest possible price, to avoid conflict with others; this behavior results in a monotonically increasing bidding curve. By contrast, when *ν* is very large, the actual bidding probability for each individual price is small, meaning that any given bid is more likely to be unique; individuals therefore tend to bid at lower prices, leading to a monotonically decreasing curve. For properly chosen values, such that the distribution curves take on an inverted-J shape, it can be seen from Fig 5 (B) that the slopes of the descending portions of the curves are essentially identical.

### The exponentially decreasing trend

The exponentially decreasing trend in the bid distribution that is mentioned above has also been recorded in other papers [7]. To ensure consistent fitting between real-world data and the model, the model should also have an exponentially decreasing component.

Next, let us discuss the density change in the model at higher bid prices. According to (4), we could get

$$\ln f(k+1)-\ln f(k)=-\lambda \left(k+1\right)+\lambda \left(k\right)+\ln\left[1-\lambda \left(k\right)e^{-\lambda \left(k\right)}\right]$$ (7)

We just take the first order approximation of Taylor expansion for the third term on the right side of Equation (7), and according to the Equation (5), we can get

$$\begin{array}{l} \ln f(k+1)-\ln f(k)\\ =-\lambda \left(k+1\right)+\lambda \left(k\right)+\ln(1)+{\frac{\left[-e^{-\lambda \left(k\right)}+\lambda \left(k\right)e^{-\lambda \left(k\right)}\right]}{\left[1-\lambda \left(k\right)e^{-\lambda \left(k\right)}\right]}|}_{\lambda \left(k\right)=0}\times \lambda \left(k\right)\\ =-\lambda \left(k+1\right)\\ =-\lambda Ce^{-\alpha \left(k+1\right)} \end{array}$$ (8)

It is obvious that *f*(k) monotonically decreases. For a given *m*(m ≫ 0), when *k* > *m*,

$$-\lambda Ce^{-\alpha \left(m+1\right)}\leq \ln f(k+1)-\ln f(k)\leq -\lambda Ce^{-\alpha V}$$ (9)

When *m* is large enough, −*λCe* ^−*α*(*m*+1)^ is nearly approaching to zero, however, −*λCe* ^−*αV*^ is a certain number less than zero, so the Equation (9) suggests that the value of ln *f*(*k* + 1) – ln *f*(*k*) exists between upper and lower bounds. The interval is narrow, so the value can be considered as approximately a constant. As a result, *f*(*k* + 1) / *f*(*k*) is approximately a positive constant less than 1. Therefore, *f*(*k*) has an approximately exponentially decreasing trend when *k* is large enough.

## Conclusions

Research concerning various types of auctions is a topic of considerable interest in various interdisciplinary fields of science. The nature of the bid distribution of LUBA and its emergence from individual psychology and behavior has aroused the interest of many experts. In this paper, in a departure from the symmetric Nash equilibrium framework, we construct a new model based on assumed information regarding the behavior of others using the Poisson distribution and an exponential distribution. This model fits the empirical data better than do models based on the symmetric Nash equilibrium because it can reproduce both the overall decreasing trend and the localized increase at lower bid prices in most cases. Furthermore, we present the effects of parameters *λ*, *α* and *ν* on the shape of the bid distribution. The impact of these three parameters on the distribution is very complex. For appropriate values of *λ*, *α* and *ν*, the distribution curve exhibits a unimodal inverted-J shape. In some cases, this curve breaks down so that it presents a monotonically increasing or decreasing function.

## Supporting Information

S1 Dataset Experimental data. There are 6 different groups of data in the file, which come from two websites. Each group includes auctions of same-valued items with nearly identical bid times. The data for groups (a) to (c) were downloaded from www.auctionair.com. The data for groups (d) to (f) were obtained from www.uniquebidhomes.com.
(XLS)
